# Effect of Remote Ischemic Conditioning in Ischemic Stroke Subtypes: A Post Hoc Subgroup Analysis From the RESIST Trial

**DOI:** 10.1161/STROKEAHA.123.046144

**Published:** 2024-02-01

**Authors:** Rolf Ankerlund Blauenfeldt, Janne Kaergaard Mortensen, Niels Hjort, Jan Brink Valentin, Anne-Mette Homburg, Boris Modrau, Birgitte Forsom Sandal, Martin Faurholdt Gude, Anne Brink Berhndtz, Søren Paaske Johnsen, David C. Hess, Claus Ziegler Simonsen, Grethe Andersen

**Affiliations:** Department of Neurology, Aarhus University Hospital, Denmark (R.A.B., J.K.M., N.H., C.Z.S., G.A.).; Department of Clinical Medicine, Aarhus University, Denmark (R.A.B., J.K.M., N.H., M.F.G., C.Z.S., G.A.).; Department of Clinical Medicine, Danish Center for Health Services Research, Aalborg University, Denmark (J.B.V., S.P.J.).; Department of Neurology, Research Unit for Neurology, Odense University Hospital, Denmark (A.-M.H.).; Department of Neurology, Aalborg University Hospital, Denmark (B.M.).; Department of Neurology, Regional Hospital Gødstrup, Denmark (B.F.S., A.B.B.).; Department of Research and Development, Prehospital Emergency Medical Services, Central Denmark Region, Aarhus, Denmark (M.F.G.).; Department of Neurology, Medical College of Georgia, Augusta University, GA (D.C.H.).

**Keywords:** cerebral small vessel diseases, humans, ischemic stroke, neuroprotection, stroke

## Abstract

**BACKGROUND:**

Remote ischemic conditioning (RIC) is a simple and noninvasive procedure that has proved to be safe and feasible in numerous smaller clinical trials. Mixed results have been found in recent large randomized controlled trials. This is a post hoc subgroup analysis of the RESIST trial (Remote Ischemic Conditioning in Patients With Acute Stroke), investigating the effect of RIC in different acute ischemic stroke etiologies, and whether an effect was modified by treatment adherence.

**METHODS:**

Eligible patients were adults (aged ≥18 years), independent in activities of daily living, who had prehospital stroke symptoms with a duration of less than 4 hours. They were randomized to RIC or sham. The RIC treatment protocol consisted of 5 cycles with 5 minutes of cuff inflation alternating with 5 minutes with a deflated cuff. Acceptable treatment adherence was defined as when at least 80% of planned RIC cycles were received. The analysis was performed using the entire range (shift analysis) of the modified Rankin Scale (ordinal logistic regression).

**RESULTS:**

A total of 698 had acute ischemic stroke, 253 (36%) were women, and the median (interquartile range) age was 73 (63–80) years. Median (interquartile range) overall adherence to RIC/sham was 91% (68%–100%). In patients with a stroke due to cerebral small vessel disease, who were adherent to treatment, RIC was associated with improved functional outcome, and the odds ratio for a shift to a lower score on the modified Rankin Scale was 2.54 (1.03–6.25); *P*=0.042. The association remained significant after adjusting for potential confounders. No significant associations were found with other stroke etiologies, and the overall test for interaction was not statistically significant (χ^2^, 4.33, *P*=0.23).

**CONCLUSIONS:**

In patients with acute ischemic stroke due to cerebral small vessel disease, who maintained good treatment adherence, RIC was associated with improved functional outcomes at 90 days. These results should only serve as a hypothesis-generating for future trials.

**REGISTRATION:**

URL: https://www.clinicaltrials.gov; Unique identifier: NCT03481777.

Remote ischemic conditioning (RIC) applied as transient cycles of limb ischemia and reperfusion by inflating and deflating a cuff on the upper extremity exerts distant organ protection in preclinical and clinical studies.^1^ RIC is a simple and noninvasive procedure that has proved to be safe and feasible in numerous smaller clinical trials.^2–5^ In the open-label randomized controlled RICAMIS trial (Remote Ischemic Conditioning for Acute Moderate Ischemic Stroke), RIC treatment initiated within 48 hours after stroke and continued for 2 weeks was associated with improved functional outcomes.^6^ This effect was most pronounced in patients with acute ischemic stroke (AIS) caused by large artery atherosclerosis (LAA).^7^ In the sham-controlled RICA trial (Chronic Remote Ischemic Conditioning in Patients With Symptomatic Intracranial Arterial Stenosis), daily RIC for 1 year was applied as a secondary preventive strategy, in patients with symptomatic intracranial stenosis. Chronic RIC significantly reduced the recurrent stroke rate in patients who were ≥50% adherent to treatment, but the trial did not meet its primary efficacy end point.^8^ In the randomized, sham-controlled RESIST trial (Remote Ischemic Conditioning in Patients With Acute Stroke), RIC was applied in the prehospital phase within 4 hours from symptom onset and repeated at the hospital. Overall, RIC was not associated with a beneficial improvement in 90-day functional outcome, but whether there is heterogeneity in treatment effect according to stroke cause and adherence is unknown.^9^

**See related article, p 880**

Here, we present a post hoc analysis of the effect of per-protocol use of RIC in subgroups of AIS and its effect on functional outcome.

## METHODS

### Study Design

The RESIST was an investigator-initiated, multicenter, randomized, patient and outcome-assessor-blinded, sham-controlled clinical trial.^9,10^ The trial was approved by Danish regional research ethics committees (ID: 1–10-72–97-17), Data Protection Agency (ID: 1–16-02–16-18), and Danish Medicines Agency (ID: 2017114177; EUDAMED: CIV-17–11-022324) as an acute study, and consent was waived in the acute prehospital phase. Consent was obtained from all patients or relatives and trial guardians as soon as possible after arrival at the hospital. The trial was registered at https://www.clinicaltrials.gov (NCT03481777) and followed the CONSORT (Consolidated Standards of Reporting Trials) reporting guideline. The data and analytical code that support the findings of this study are available from the corresponding author upon reasonable request. Individual participant data that underlie results in this article will be shared after deidentification. To gain access, data requestors will need to sign a data processing agreement. Eligible patients were adults (aged ≥18 years), who were independent in activities of daily living (modified Rankin Scale [mRS] score ≤2), with a prehospital putative stroke (evaluated by prehospital stroke score), presenting within 4 hours from symptom onset. Patients with diagnoses of AIS or intracerebral hemorrhage were defined as the target population. Patients were randomized 1:1 to RIC or sham. RIC devices and sham devices were programmed to perform 5 cycles, each with 5 minutes of cuff inflation and 5 minutes with a deflated cuff. For the RIC device, the minimum cuff pressure was 200 mm Hg; if the initial systolic blood pressure was above 175 mm Hg, the cuff would inflate to 35 mm Hg above the systolic blood pressure to ensure complete arterial occlusion (maximum cuff pressure, 285 mm Hg). The sham device would only inflate to a pressure of 20 mm Hg. At 6 hours after completion of the first RIC/sham protocol, an additional series of RIC/sham was performed (postconditioning) for all patients with a target diagnosis. Treatment was started immediately after randomization in the ambulance or helicopter. In patients with a target diagnosis admitted to Aarhus University Hospital, ischemic postconditioning was continued twice daily for 7 days.

If the patient was discharged before the seventh day, he/she would administer the RIC/sham treatment at home and subsequently return the device. Data on compliance was stored on each device. The protocol has been described in detail elsewhere.^9,10^

### Adherence

Adherence data were electronically stored on each RIC and sham device and consisted of the number of cycles and time stamps for each completed cycle. Data were downloaded upon return of each device to the stroke center. Acceptable treatment adherence was defined as at least 80% of planned RIC cycles received. For patients planned to receive acute and 6-hour treatment, 8 of 10 cycles would be defined as acceptable treatment adherence and similar when 56 of 70 cycles were received in patients with planned RIC/sham for 7 days. In the analysis, adherent RIC patients are compared with sham-treated patients with the same level of adherence.^11^

### Outcomes Measures

The primary efficacy outcome measure was the mRS (ranging from 0 to 6: 0: no symptoms and 6: dead) at 90 days in patients with acceptable adherence in different AIS subgroups. The blinded assessments could be in-person or telephone-based assessments and were performed by at least 2 independent assessors. In the case of rater disagreement, a third assessor (in-person or telephone) would act as the final assessor. National Institutes of Health Stroke Scale (NIHSS) was performed at hospital arrival and after 24 hours in the target population. AIS subgroups were based on the clinical impression at discharge by the treating physician and were performed according to the TOAST (Trial of ORG 10172 in Acute Stroke Treatment) criteria.^12^ AIS subgroups were LAA disease (symptomatic precranial or intracranial stenosis; *International Classification of Diseases*, *Tenth Revision* [*ICD-10*]: I63.2, I63.0, I63.1, and I63.5), small vessel disease (SVD; *ICD-10*: I63.3), cardioembolic (*ICD-10*: I63.4), unknown/multiple/rare (*ICD-10*: I63.8 and I63.9), or missing.

### Statistical Analysis

The analysis was performed using the entire range (shift analysis) of the mRS (ordinal logistic regression), with a random effect on the stratification groups as previously described in the target population group.^9^ To investigate the effect of stroke cause and treatment effect on the primary end point, we used an ordinal logistic regression model, relating the odds of the primary outcome with the covariate of interest and the randomization group (RIC/sham) in patients with an acceptable treatment adherence of at least 80%.

Adjustments for reperfusion therapy (yes/no), age (continuous), women/men, prestroke mRS (categorical [mRS, 0, 1, 2]), and prehospital stroke severity (prehospital stroke score ranges from 1 to 6) were performed as a post hoc analysis to explore the effect of potential confounders. No correction for multiple testing has been made, as the analysis is only for hypothesis generation purposes. The overall test for interaction between treatment and stroke cause was performed using the χ^2^ test. All analyses were performed with a 2-sided alpha level of 0.05. Statistical analyses were performed in STATA SE, version 18.0 (StataCorp, College Station, TX).

## RESULTS

From March 16, 2018, to November 11, 2022, a total of 1611 patients underwent prehospital screening. Of these, 111 were screen failures. A total of 1500 patients were randomized; 67 patients were excluded mainly due to withdrawal of consent (Figure S1) A total of 902 patients were included in the target population group, and of these, 737 (82%) patients had AIS and 165 (18%) had ICH. In 39 patients with transient neurological symptoms and an acute lesion on MRI, there was no available information on stroke cause. In the remaining 698 patients with AIS, 147 (21%) had symptomatic precerebral or intracranial LAA disease, 93 (13%) had stroke due to SVD, 101 (15%) had cardioembolic, and 357 (51%) had multiple identified causes, rare causes, or undetermined causes. Baseline characteristics were well balanced between the 2 treatment arms. However, among patients with cardioembolic stroke, 37 (80%) in the RIC group had AF versus 33 (60%) in the sham group (Table S1; *P*=0.027). In 452 of 698 patients with AIS with an acceptable adherence, baseline characteristics are presented in Table 1.

**Table 1.**
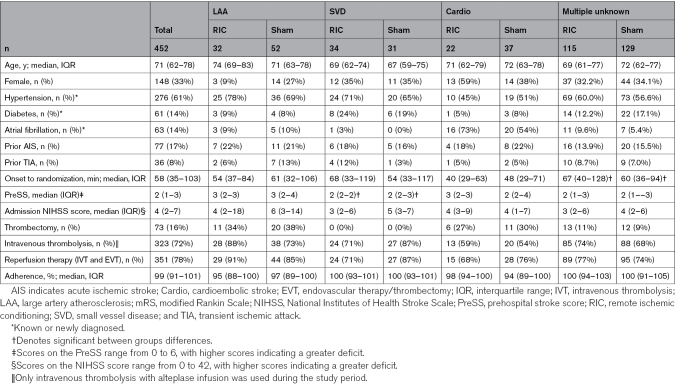
Baseline Demographic, Clinical, and Treatment Characteristics Stratified by Stroke Cause in Patients With a Good Treatment Adherence

### Functional Outcome

In patients with a predefined acceptable treatment adherence (≥80%), RIC was associated with improved functional outcomes in patients with AIS due to SVD (odds ratio, 2.54 [95% CI, 1.03–6.25]; *P*=0.042). The adjusted analysis did not change the association (adjusted odds ratio, 3.58 [95% CI, 1.30–9.88]; *P*=0.013).

There was no significant effect of adherence to RIC treatment and 90-day functional outcome in other subgroups of AIS (Table 2). Between-group distribution of the 90-day mRS in all subgroups is presented in Figure S2.

**Table 2.**
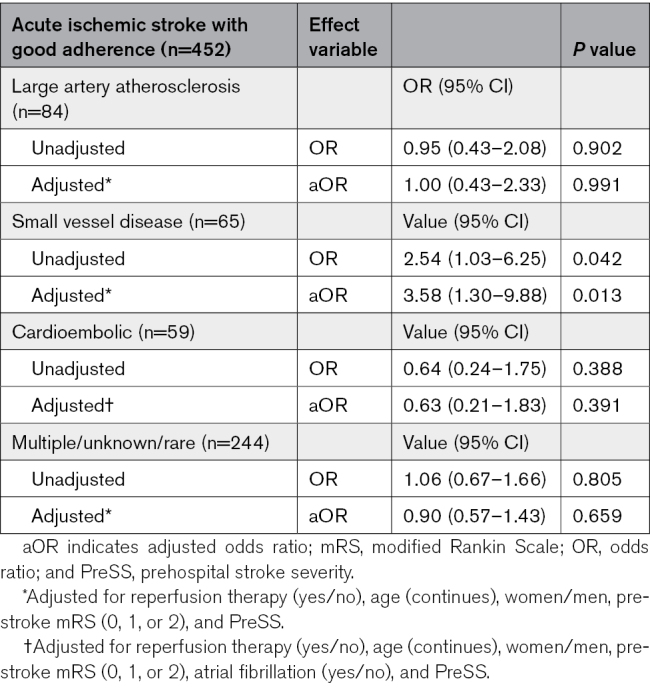
Odds for Improvement in Functional Outcome in Acute Ischemic Stroke Subtypes With Good Treatment Adherence

Overall, a favorable functional outcome (mRS, 0–2) was found in 373 (83%) of 452 patients with AIS. RIC treatment was not associated with more patients achieving a favorable functional outcome in any of the subgroups (Table 3). No significant differences were found in early markers of neurological improvement or functional outcome (Table 3). The overall test for interaction between treatment and stroke cause was not significant (χ^2^, 4.33 [df, 3]; *P*=0.23).

**Table 3.**
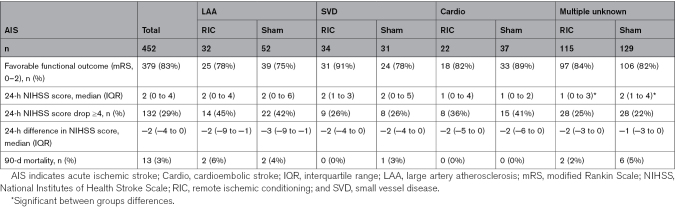
Early and Long-Term Neurological Outcomes in Subgroups of Acute Ischemic Stroke With Good Treatment Adherence

The Figure shows a forest plot illustrating the odds ratio for a shift toward a better outcome stratified by treatment duration, RIC, and acceptable adherence in different AIS etiologies. In patients with SVD, receiving 80% of 7 days of RIC treatment was associated with a significant improvement in functional outcome but not in patients who planned to receive only the acute and 6-hour treatment. For other stroke etiologies, there was no significant benefit of receiving additional treatment for 7 days. The overall test of interaction between treatment and stroke cause was not statistically significant (acute and 6-hour treatment: χ^2^, 2.09 [df, 3]; *P*=0.55 and 7 days: χ^2^, 3.31 [df, 3]; *P*=0.35).

**Figure.**
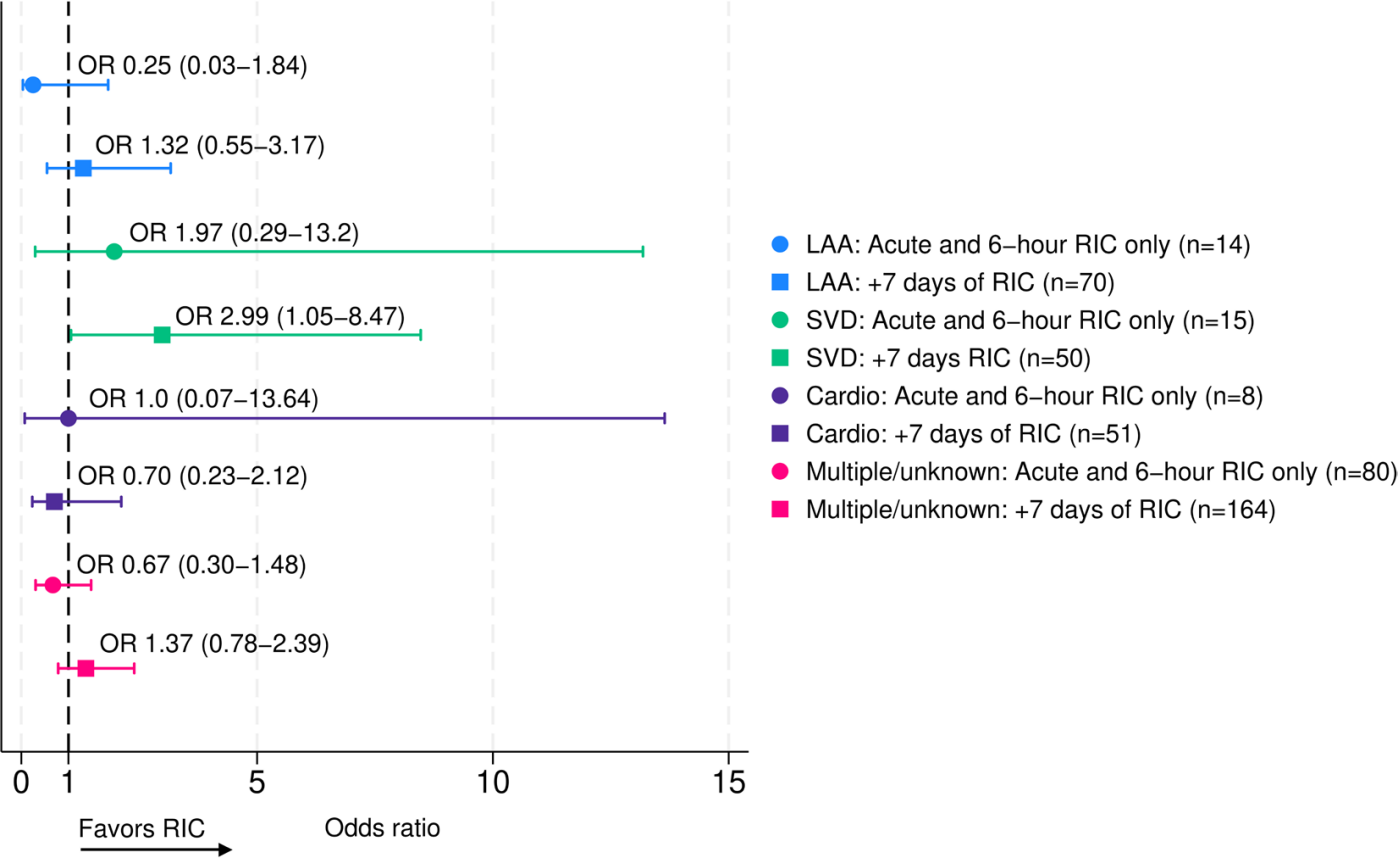
**The effect of treatment duration on functional outcome in acute ischemic stroke subgroups.** ORs are unadjusted. Cardio indicates cardioembolic stroke; mRS, modified Rankin Scale; OR, odds ratio; LAA, large artery atherosclerosis; RIC, remote ischemic conditioning; and SVD, small vessel disease.

## DISCUSSION

In this per-protocol subgroup analysis, we found evidence of treatment heterogeneity of RIC within stroke subgroups. In patients with AIS due to SVD and acceptable treatment adherence, RIC treatment compared with sham was associated with a significant improvement in mRS at 90 days. We found no association with other AIS etiologies. These results may guide future studies on RIC in stroke due to SVD but should be interpreted with caution.

The primary results of the RESIST trial did not show a treatment benefit of combined prehospital and in-hospital RIC for AIS. Two recent randomized trials applied RIC at later time points and demonstrated the possible efficacy of RIC. In the nonsham controlled trial RICAMIS (N=1893), bilateral upper extremity RIC was initiated within 48 hours of symptom onset in patients with moderate to severe AIS (NIHSS score, 6–16), who were not treated with reperfusion therapy. The majority of patients in RESIST received reperfusion therapies (75%). The median time from onset to RIC was 25 hours in RICAMIS compared with 56 minutes in RESIST. In the sham-controlled RICA trial (N=3033), bilateral upper extremity RIC was applied as a daily treatment for 1 year following a recent qualifying stroke/TIA. The primary end point of time to first recurrent ischemic stroke was not reached, but in patients who were adherent to treatment (defined as adhering >50% of days), daily RIC significantly reduced the risk of recurrent stroke.

In RESIST, the unilateral dosing of RIC may be insufficient to elicit a response in a comorbid stroke population, and RIC may not provide enough protection in the hyperacute phases of stroke as the positive trials that have applied RIC in the subacute or chronic phases of stroke. On the other hand, a placebo response in the RICAMIS trial cannot be ruled out because the trial was not sham-controlled and the mRS is not an entirely objective measure.^11,13^

Recently, the RICAMIS authors published a subgroup analysis demonstrating a higher treatment effect of RIC in patients with AIS due to LAA compared with non-LAA.^7^ It is unknown how many patients had a stroke due to SVD, which also may be limited by the inclusion criteria of stroke severity (NIHSS) of at least 6. Previous smaller trials have tested long-term RIC in patients with SVD or intracranial stenosis and found reduced growth in white matter hyperintensity volume compared with the control group and improved long-term cognitive function.^14,15^ The protective mechanisms may involve reduced inflammatory responses, enhanced recovery, and angioneogenesis.^1,16–20^ Another study found beneficial effects on administrating isosorbide mononitrate in patients with SVD.^21^ Interestingly, nitric oxide release is one of the proposed mechanisms of RIC and may explain some of the observed differences.^1^ It is currently unknown whether these molecular pathways and the effects of RIC are differentially expressed in stroke subtypes.

In the present study, adherent patients with AIS caused by SVD had significantly improved outcomes when treated with RIC. The associations remained significant and strengthened after adjusting for potential confounders. In the stratified analysis according to the planned duration of RIC (acute and 6 hours versus additional 7 days), the direction of the estimates both favored RIC, but only the 7-day RIC treatment was associated with increased odds for functional improvement. The effect of RIC may be more pronounced if given for a longer duration or when applied after the acute phase of stroke, which would be in line with the results from the RICA and RICAMIS trials. The study has several limitations. The classification of stroke etiologies was based on the clinical impression of the discharging physician and was not centrally adjudicated. The sample size was small, the stroke severities at baseline were mild to moderate (NIHSS score, 4), and many received reperfusion therapies (75%), factors that risk introducing a ceiling effect to any potential treatment benefit of RIC. This may also limit the sensitivity secondary end points, such as a 24-hour drop in NIHSS of at least 4 points.

The current results should be interpreted with caution, as the primary trial result was neutral, and the subgroup of patients with SVD (n=93) was small. Furthermore, no correction for multiple comparisons has been made in this post hoc analysis, and the results should only serve as hypothesis-generating and guide future trials on RIC treatment in patients with AIS.

## CONCLUSIONS

In this post hoc subgroup analysis, we found that in adherent patients with AIS due to SVD, RIC was associated with a significant improvement in mRS at 90 days. We found no significant association between other etiologies of AIS and functional outcomes.

## ARTICLE INFORMATION

### Acknowledgments

The data that support the findings of this study are available from the corresponding author upon reasonable request. Individual participant data that underlie results in this article will be shared after deidentification. Proposals should be directed to rolfblau@rm.dk. To gain access, data requestors will need to sign a data processing agreement. Dr Blauenfeldt and J.B. Valentin had full access to all the data in the study and took responsibility for the integrity of the data and accuracy of the data analysis.

### Sources of Funding

The trial received funding from TrygFonden (120636), the National Instituted of Health (1R01NS112511-01A1), the Novo Nordisk Foundation (NNF00052924 and NNF0060998) the Manufacturer Vilhelm Pedersen and Wife’s Foundation (NNF16OC0023474), and the Aase and Ejnar Danielsens Foundation (10–002120). The funder had no role in the design and conduct of the study; collection, management, analysis, and interpretation of the data; preparation, review, or approval of the article; and decision to submit the article for publication.

### Disclosures

Dr Blauenfeldt received lecture fees from Bayer, Pfizer, and Novo Nordisk, unrelated to the submitted work. Dr Hess was supported by a research grant from the National Institutes of Health. Dr Simonsen was supported by research grants from the Health Research Foundation of the Central Denmark Region. The other authors report no conflicts.

### Supplemental Material

Figures S1–S2

Table S1

CONSORT Checklist and Protocols

## Supplementary Material


